# Efficacy, safety, and cost of surgical versus nonsurgical treatment for carpal tunnel syndrome: A systematic review and meta-analysis: Retraction

**DOI:** 10.1097/MD.0000000000006778

**Published:** 2017-04-21

In the article, “Efficacy, safety, and cost of surgical versus nonsurgical treatment for carpal tunnel syndrome: A systematic review and meta-analysis”,^[1]^ which appeared in Volume 95 issue 40 of *Medicine*, a reader brought to our attention a mistake in the publication. After further investigation, it was found that the authors’ analysis of surgical and nonsurgical treatment was incorrect.

The authors explained: “In our recent paper, we reported a systematic review and meta-analysis to compare the clinical efficacy, safety, and cost of surgical versus nonsurgical intervention. In the course of the data analysis, we discovered that nonsurgical treatment is more effective than surgical treatment. However, we made mistakes in conducting the forest plots of primary outcome about clinical efficacy and subgroup analysis, which resulted in getting the results backwards and getting the opposite conclusion.”
